# Repeated exposure to task-relevant and task-irrelevant information – and their interaction – affect visual search performance

**DOI:** 10.3758/s13414-025-03217-0

**Published:** 2026-03-10

**Authors:** Chloe Callahan-Flintoft, Patrick H. Cox, Emma M. Siritzky, Stephen R. Mitroff, Kelvin S. Oie, Dwight J. Kravitz

**Affiliations:** 1 U.S. Army DEVCOM Army Research Laboratory, Aberdeen Proving Ground, Adelphi, MD USA; 2 Lehigh University, Bethlehem, PA USA; 3 The George Washington University, Washington, DC USA

**Keywords:** Attention, Visual search, Selection history, Task exposure

## Abstract

The human visual system adapts to statistical regularities in the environment to facilitate visual processing. While laboratory-based tasks make clear distinctions between how task-relevant and task-irrelevant visual information can guide this adaptation, such discretization is rarely available in the real world. As such, it remains unclear exactly what information the visual system tracks to flexibly adapt to a given task. The current study used a massive visual search dataset from the mobile game *Airport Scanner*. Effects of exposure over a range of more task-relevant (e.g., target presence) to less task-relevant (e.g., background context) features were analyzed in an omnibus model to predict response times in both target-present and target-absent trials. As in previous work (Kramer et al., *Journal of Experimental Psychology: General*, 151 (8), 1854, 2022), increased exposure to target-present trials significantly sped up the detection of targets and slowed the rejection of target-absent trials. Exposure to salient distractors reduced response times for target-present trials, potentially as a result of learned distractor suppression (Gaspelin & Luck, *Trends in cognitive sciences*, 22 (1), 79-92, 2018) or increased familiarity (Mruczek & Sheinberg, *Perception & psychophysics*, 67 (6), 1016-1031, 2005), but had no effect on target-absent trials. Exposure to background information decreased response times in both target-present and target-absent trials, with notable interactions between target and background exposure. Specifically, the effect of background information was more pronounced when target exposure was low, suggesting that less task-relevant context information is more likely to be tracked in the absence of more task-relevant information, namely, the presentation of targets. The findings highlight the importance of considering multiple sources of exposure in visual search tasks and demonstrate the value of large datasets in quantifying their complex interactions.

## Introduction

The effects of previous exposure are readily observed in visual search tasks (e.g., Adam & Serences, 2021; Theeuwes et al., 2022). These effects are interpreted differently (e.g., priming, carryover effects, learning) dependent upon differences in task-defined goals and dynamics, but they imply abilities of the human visual system to leverage previous exposures to relevant features of the task environment to shape future behavioral response. In the laboratory, such abilities are often explored by carefully controlling and manipulating the statistical regularities of experimental tasks and conditions, which are operationally defined as either task-relevant or task-irrelevant (e.g., Jones & Kaschak, 2012; Sauter et al., 2021). However, while this discretization can enable theoretical inference, in natural environments such clear distinctions are rarely, if ever, possible. Real-world environments are typically visually dense and dynamic, making inferences about what combinations of environmental features are causal in visual search behavior challenging. Moreover, task goals can also change, creating concomitant changes in the relevance of previously important environmental features over time. As such it is a non-trivial problem to determine what statistical regularities should be tracked to improve task performance.

In cognitive psychology, the effect of previous trials on the current trial’s performance has been either explored as a topic of direct interest (e.g., contextual cueing (Chun, 2000), serial dependence (Fischer & Whitney, 2014; Zhang, & Lewis-Peacock, 2024), learned distractor suppression (Britton & Anderson, 2020; Wang & Theeuwes, 2018)) or controlled to eliminate confounds (e.g., Greenwald, 1976). Exposure effects are found in multiple stages of task execution (e.g., motor response execution, response selection, visual perception, and the deployment of attention). At the motor response level, motor priming is characterized as the repetition of a given motor response, speeding up one’s ability to generate that response subsequently whilst simultaneously inhibiting other responses (e.g., Rosenbaum & Kornblum, 1982; Smith et al., 2008). Even the selection of that motor response has been found to be facilitated when the same response was selected on previous trials (Kiesel et al., 2007). More broadly, carryover effects have been shown to not only affect the selection and generation of a response to stimuli but also influence visual processing. Many models of attentional deployment have primarily focused on the influences of task-related, top-down goals (e.g., Folk et al., 1992; Leber & Egeth, 2006; Luque et al., 2021) and stimulus-driven, bottom-up signals (e.g., Bruce & Tsotsos, 2009; Theeuwes, 1992) as well as the interaction between the two (e.g., Gaspelin et al., 2016; Zelinsky et al., 2005). However, a third factor, selection history, is increasingly featured as a driver of visual search performance (Theeuwes, 2019). Selection history is often used as an umbrella term that encompasses effects such as past rewards (e.g., Failing & Theeuwes, 2014; Libera & Chelazzi, 2006) and learned statistical regularities (e.g., Chun & Jiang, 1998). This factor stands apart from traditional views on top-down and bottom-up drivers of attention as studies on selection history show that attention can be guided towards or away from previously experienced objects even though these objects are not the most salient in a scene nor included in the current task set.

Carryover effects have been shown across a range of task relevance. Previous work demonstrated this by using big data from the mobile game *Airport Scanner* (Kramer et al., 2022). In the game, participants act as airport security screeners, searching bags one by one for prohibited items (targets) among allowed items (distractors) (Kedlin, Co.). A significant effect of target and salient distractor exposure was found on current trial performance. This supported other findings which showed the visual system’s ability to track ensemble statistics of distracting information (Hansmann-Roth et al., 2021; Oriet & Hozempa, 2016). Critically, in the previous work using *Airport Scanner*, there was also an effect of background information exposure (Kramer et al., 2022). Specifically, the more previous exposure a participant had to the given bag type (task-irrelevant background information with no predictive relationship to the presence or absence of targets) presented on the current trial, the faster they were at locating the target. This finding underscores that there is a variety of information that provides context for a given task. Importantly, here the characterization of information as “task-irrelevant” is given from the perspective of the experimenter. In other words, information is referred to as task-irrelevant when the experimenter does not intend for that information to be used by the participant to complete the experimental task, i.e., there is no statistical relationship to the target.

While a number of studies have looked at both target (Talcott et al., 2022; Wang et al., 2023; Witkowski & Geng, 2019) and distractor (Kim et al., 2023; Sauter et al., 2021) exposure effects, these effects are often modeled individually. It is therefore unclear whether these different types of exposure effects account for overlapping portions of variance in performance, whether some forms of exposure have larger effects on performance than others (e.g., exposure to task-relevant information (e.g., targets) having a greater effect than task-irrelevant information (e.g., background bag type)), and whether or not these effects interact with one another. The current work used the same *Airport Scanner* gameplay dataset to combine target exposure, salient distractor exposure, and bag type exposure in a single omnibus linear model. First, the model was fit and applied to Level 1 of the game (the same data used in the previous work), and then, to demonstrate robustness, the model was also fit and applied to data from the preceding practice level. Together, these models demonstrate that exposure to task-relevant and -irrelevant information accounts for unique portions of variance in performance and has interaction effects, providing insights into what information the visual system keeps track of to facilitate task execution.

## Methods

### Participants

Participants in this study were anonymous users of the mobile application *Airport Scanner* (Kedlin Co.). Data collection was approved by The George Washington University Institutional Review Board. Here, “participant” refers to each unique device ID, assigned when the application was downloaded onto a device. Because the data are anonymous, it is not possible to know if each device was used by a single unique participant. As such, it is possible that more than one person played the game on a given device or that the same person played the game on multiple devices.

The model was run on two subsets of gameplay, described in more detail in the *Procedure* section. *Level 1* refers to the model being fit to the first ten bags of Level 1, and *Practice* refers to the model being fit to the last ten bags of the practice levels. After data selection (described in the *Procedure* section), 292,188 participants were used to fit the Level 1 model to hit trials and 196,416 were used to fit the model to correct rejection trials. In Practice, 506,001 participants were used to fit the model to hit trials and 328,799 participants were used to fit the model to correct rejection trials. Data selection criteria, along with the analysis plan, were preregistered (https://osf.io/3rka8/). Contact the authors to request access to the dataset.

### Procedure

**Fig. 1:**
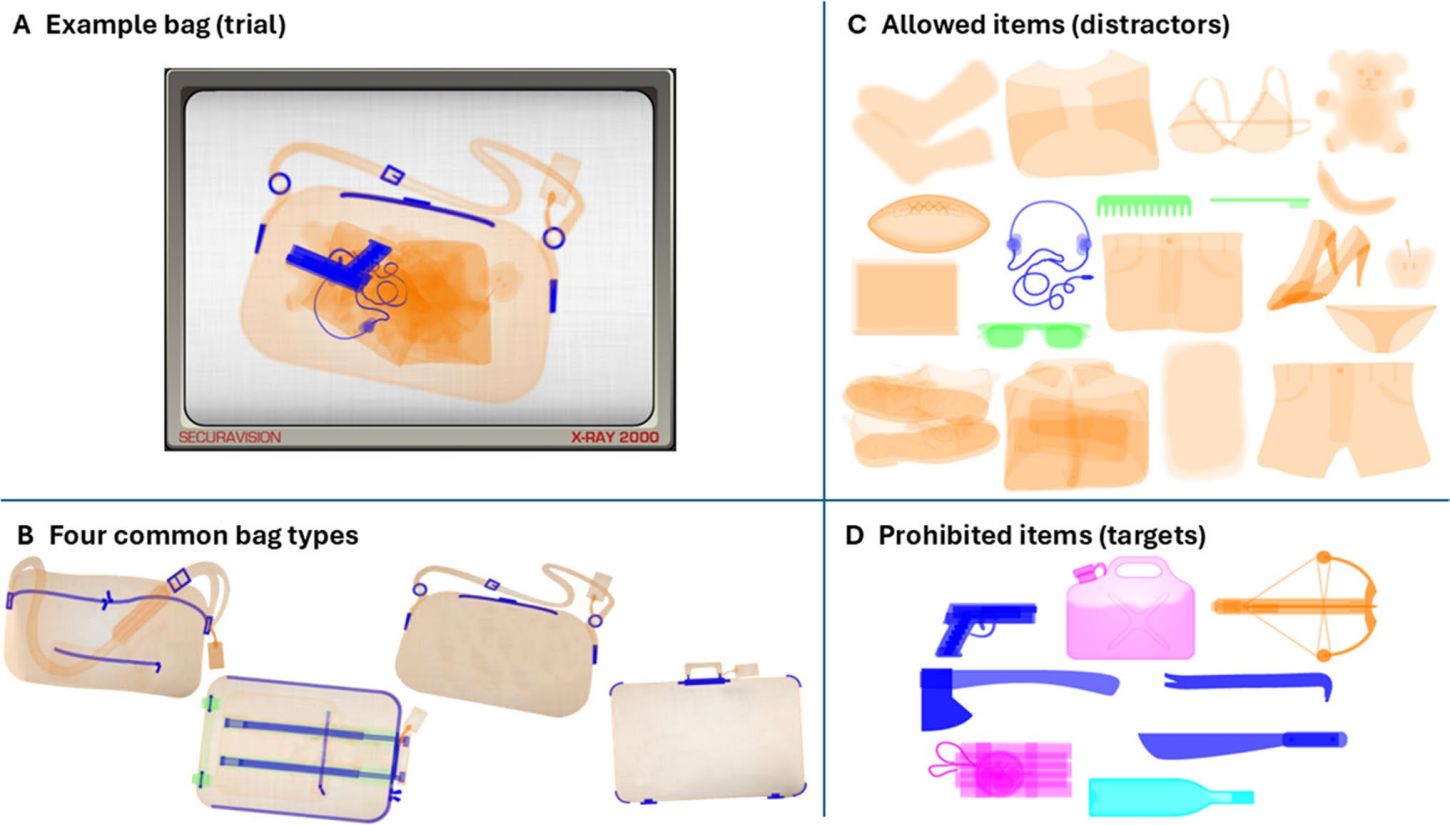
Example stimuli from Airport Scanner. (**A**) Screenshot of an example trial. This example is a target-present trial (the gun) and a salient distractor-present trial (the headphones). (**B**) The four bag types present in the levels of gameplay used in this analysis. These four bag types were the only ones present in the practice level and the only ones that could contain multiple items in level 1 (two other infrequent level-specific bags contained no distractors and either contained or did not contain a target). (**C**) Allowed items, considered here as distractors as they are not what participants are searching for. (**D**) Prohibited items, considered here as targets as participants must locate them and respond to them (tap with finger)

*Airport Scanner* is a mobile game where participants play the role of airport security screeners. The game is a visual search task where participants monitor bags as they move along a conveyor belt of a simulated x-ray scanner. Participants tap on prohibited items (targets) that may appear in bags among allowed items (distractors) (Fig. 1). To move bags along the conveyor belt faster, and receive bonus points, participants can tap on a bag and swipe it across the screening to proceed more quickly through the level. Thus, for target-present bags, participants must tap the targets as quickly and as accurately as possible, whereas in target-absent bags, participants must swipe the bag along the conveyor belt as fast as possible in order to earn the maximum number of points. Participants receive visual feedback after each bag as well as auditory feedback if the game is unmuted on the player’s device.

The model was fit to data from two different points in the game. The first was Level 1 (the *Honolulu* airport, which is the first airport participants move to after two *Trainee* levels), as this is the same section of gameplay used in the previous study. Specifically, the first ten trials of Level 1 were used. Then, the model was fit again to the last ten trials of Practice (the second *Trainee* level preceding Level 1).

In both sections, ten trials were used to fit the model. The first nine trials were the exposure trials used to predict performance on the tenth trial. This means that every participant contributed a single point of data: their exposure score for the three variables of interest over the first nine trials (the independent variables), and the log response time of their tenth trial (the dependent variable). Exposure to information was quantified in three predictors of the omnibus model: target exposure, salient distractor exposure, and bag type exposure. Exposure was defined as the binomial z-score,

$$z={(\mathrm{p}-\mu)}/\sqrt(\mathrm{p}^\ast\left(1-\mathrm{p}\right)$$

where *p* is the observed proportion of exposure trials containing the factor for a given participant and μ is the average prevalence rate of the factor across participants. For target exposure, this proportion was defined as the number of exposure trials that were target-present trials divided by the total number of exposure trials (9). Target trials had a μ = 0.62 prevalence in Practice and μ = 0.57 prevalence in Level 1. Salient distractor exposure was calculated using the proportion of exposure trials that contained the *headphones* item. The *headphones* were chosen as the salient distractor as they are dark blue, whereas the other distractors available during these early levels of the game are mostly orange with some green (Fig. 1C). Notably, the *headphones* are also the same color as many of the targets that are dark blue as well (Fig. 1D). This predictor was chosen as it represents stimuli that may not be directly task relevant, in that the presence of a salient distractor did not correlate with the presence of a target, but may still have captured participants’ attention or required them to ignore or reject the salient distractor in trials where it appeared. It is therefore expected that this variable will account for a unique portion of variance, such that it remains a significant predictor of performance even when modeled in combination with target and bag exposure. In the game, distractors for each trial were sampled with replacement from the pool of available distractors for a given level. *Headphones* had a μ = 0.32 prevalence in Practice and μ = 0.26 prevalence in Level 1. Finally, bag type exposure was calculated from the proportion of exposure trials that had the same bag type as the bag type present in the performance trial. There were four bag types: *briefcase*, *carryon*, *purse*, and *duffle* (Fig. 1B)*.* Participants were only included in analysis if one of these four bags was present on the performance trial. These four bags were used as they were the only bags used in Practice, and in Level 1 they were the only bag types that could contain multiple items (Level 1 contained two other bag types that could only have one (a target) or zero items). The four common bag types had a μ = 0.25 prevalence in Practice and a μ = 0.22 prevalence in Level 1. The dependent variable used here was response time, operationalized as the first time the bag was either tapped or the time it took for the bag to be swiped across the screen.

#### Data analysis

As this dataset was generated from a mobile game and not an experimental paradigm, a number of decisions were made in terms of selection criteria for participants used and what section of gameplay was analyzed in order to address the research question at hand. A full list of criteria is given in the preregistration (https://osf.io/3rka8/). Specifically, participants were only included in the analyses if they played straight through the practice levels (*Trainee* levels 1 and 2) and the first ten trials of Level 1 (*Honolulu Day 1*) without repeating any levels, completing any interim tasks in the game, or activating any upgrades to ensure that all participants had an equal amount of experience with the game. Participants were only included in the analyses if, on the performance trial (the tenth trial), the salient distractor (i.e., *headphones*) was present in order to test how exposure to the salient distractor affected performance on trials with that distractor. In addition to only including participants who saw one of the four main bag types on the performance trial, another selection criteria was that there were more than four distractors present in the performance trial bag, as the current work was interested in studying processes involved in visual search and therefore required some presence of interfering information. In order to make all target-present performance trials comparable, only participants who were presented a maximum of one target on the performance trial and responded accurately in the performance trial were included. Finally, to improve data quality as there was no experimental control in terms of how participants engaged with the game (i.e., people could play the game in a variety of noisy or otherwise distracting environments), participants were only included if their response times on the performance trial were between 250 and 10,000 ms. This criterion led to the exclusion of ~4% of participants.

To quantify exposure to target presence, the salient distractor, and bag type, a binomial z-score (described above) was used. These three binomial z-scores were then entered into a linear model using the *fitlm* function in MATLAB (Mathworks Inc., Natick, MA, USA) to predict log-transformed response times on the performance trials. All beta estimates therefore are in log seconds. Only accurate trials were modeled with hit (target-present), and correct rejection (target-absent) trials were analyzed separately.

## Results

### Level 1

**Fig. 2:**
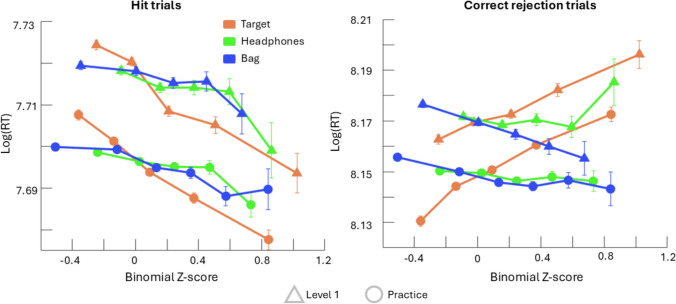
Average log response time as a function of exposure binomial z-score to target presence (orange), headphones (green), and bag (blue). Hit trials plotted on the left with correct rejection trials on the right. Level 1 means are plotted as triangles while Practice means are plotted as circles. Error bars denote standard error. For all graphs, binomial z-score range was trimmed to make all factors comparable and bins with less than 1,000 people were excluded from plotting

Fitting the model to Level 1 data showed that for hit trials there was a significant effect of target exposure (β = −0.03, *p* = 2.20 x 10^−35^) and headphones exposure (β = −0.01, *p* = 2.28 x 10^−7^) on response time (Fig. 2). There was no effect of bag type exposure (β = −0.002, *t* = −1.26, *p* = 0.21), nor were any of the interaction terms significant (Table 1).

**Table 1:** All factors and interactions and associated statistics in the linear model fitted to hit trials of Level 1. Difference in AIC scores between the full model (all predictors and interactions included) and a model with that predictor excluded is listed for all predictors in the rightmost column. Positive values indicate a better model fit when that predictor is included

Level 1: hit trials	Estimate	SE	t-stat	*p*-value	Δ AIC
Target	**−0.03**	**0.002**	**−12.90**	**4.42 x 10**^**−38**^	**164.45**
Headphones	**−0.01**	**0.002**	**−5.17**	**2.28 x 10**^**−7**^	**24.78**
Bag	−0.002	0.002	−1.26	0.21	−0.42
Target × Headphones	0.01	0.01	0.91	0.36	−1.18
Target × Bag	0.01	0.01	0.84	0.40	−1.30
Headphones × Bag	0.001	0.01	0.29	0.77	−1.92
Target × Headphones × Bag	−0.006	0.02	−0.30	0.76	−1.91

For correct rejection trials, target exposure (β = 0.02, *p* = 4.54 x 10^−19^) and bag type exposure (β = −0.02, *p* = 8.80 x 10^−14^) had significant effects on response time. There was a significant interaction between target and bag exposure (β = 0.02, *p* = 0.02). There was no effect of headphones exposure (β = −0.002, *p* = 0.43), nor were any of the other interaction terms significant (Table 2).

**Table 2:** All factors and interactions and associated statistics in the linear model fitted to correct rejection trials of Level 1

Level 1: correct rejection trials	Estimate	SE	t-stat	*p*-value	Δ AIC
Target	**0.02**	**0.003**	**8.92**	**4.54 x 10**^**−19**^	**77.62**
Headphones	−0.002	0.003	−0.79	0.43	−1.38
Bag	**−0.02**	**0.003**	**−7.46**	**8.80 x 10**^**−14**^	**53.62**
Target × Headphones	0.003	0.01	0.39	0.69	−1.84
Target × Bag	**0.02**	**0.01**	**2.28**	**0.02**	**3.21**
Headphones × Bag	0.01	0.01	0.64	0.52	−1.59
Target × Headphones × Bag	0.003	0.03	0.09	0.93	−1.99

### Practice

**Fig. 3:**
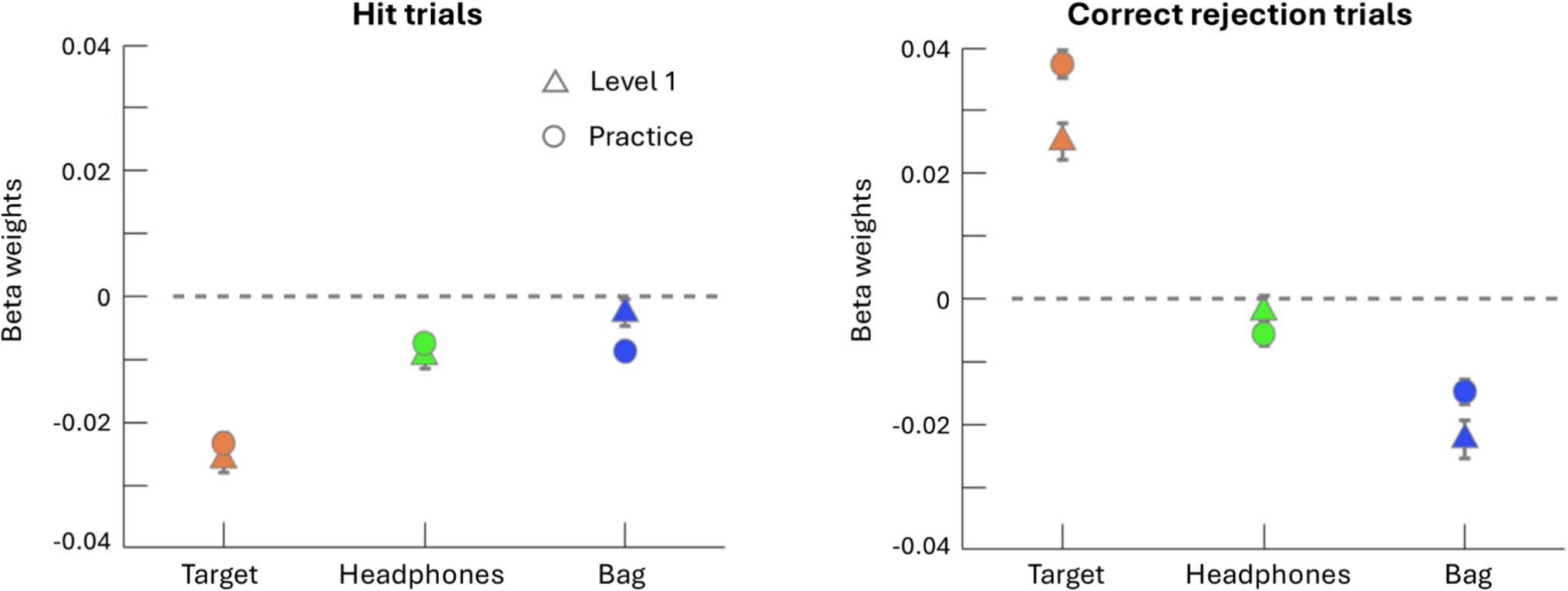
Beta weights for the three main factors in the linear model fit to Level 1 (triangle) and Practice (circle) data

Fitting the model to Practice data revealed a similar pattern to that of Level 1 in that in hit trials there were significant effects of target exposure (β = −0.02, *p* = 1.32 x 10^−49^) and headphones exposure (β = −0.01, *p* = 1.33 x 10^−8^) (Fig. 3). However, in Practice, bag type exposure also had a significant effect on response time (β = −0.01, *p* = 1.89 x 10^−9^) and there was a significant interaction between target exposure and bag type exposure (β = −0.03, *p* = 3.83 x 10^−10^), unlike in hit trials of Level 1 (Table 3).

**Table 3:** All factors and interactions and associated statistics in the linear model fitted to hit trials of Practice

**Practice: hit trials**	Estimate	SE	t-stat	*p*-value	Δ AIC
Target	**−0.02**	**0.002**	**−14.81**	**1.32 x 10**^**−49**^	**217.25**
Headphones	**−0.01**	**0.001**	**−5.68**	**1.33 x 10**^**−8**^	**30.29**
Bag	**−0.01**	**0.001**	**−6.01**	**1.89 x 10**^**−9**^	**34.08**
Target × Headphones	−0.002	0.004	−0.38	0.70	−1.85
Target × Bag	**0.03**	**0.005**	**6.26**	**3.83 x 10**^**−10**^	**37.20**
Headphones × Bag	0.0005	0.004	0.12	0.90	−1.98
Target × Headphones × Bag	−0.01	0.01	−1.12	0.26	−0.75

In correct rejection trials of Practice (Table 4), as with Level 1, there was a significant effect of target (β = 0.04, *p* = 2.74 x 10^−66^), bag exposure (β = −0.01, *p* = 2.23 x 10^−13^), and a significant interaction between target and bag type exposure (β = 0.05, *p* = 2.64 x 10^−9^).Additionally, the main effect of headphones exposure was significant (β = −0.01, *p* = 2.09 x 10^−3^).

**Table 4:** All factors and interactions and associated statistics in the linear model fitted to correct rejection trials of Practice

Practice: correct rejection trials	Estimate	SE	t-stat	p-value	Δ AIC
Target	**0.04**	**0.002**	**17.20**	**2.74 x 10**^**−66**^	**293.79**
Headphones	**−0.01**	**0.002**	**−3.08**	**2.09 x 10**^**−3**^	**7.47**
Bag	**−0.01**	**0.002**	**−7.33**	**2.23 x 10**^**−13**^	**51.79**
Target × Headphones	0.01	0.006	1.46	0.14	0.14
Target × Bag	**0.06**	**0.006**	**8.73**	**2.61 x 10**^**−18**^	**74.16**
Headphones × Bag	−0.01	0.005	−1.37	0.17	−0.13
Target × Headphones × Bag	0.01	0.02	0.76	0.45	−1.43

### Interaction between target and bag exposure

**Fig. 4:**
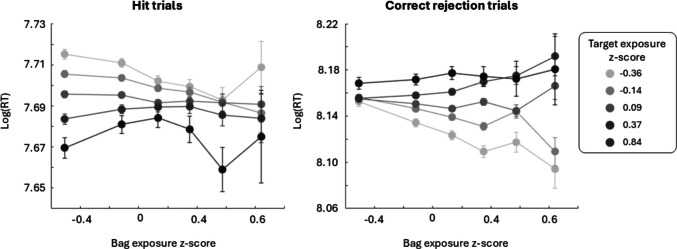
Average log response times for Practice trials, plotted as a function of bag exposure z-scores at various levels of target exposure z-scores (darker dots representing more previous exposure to target-present trials) for hit (**left**) and correction rejection (**right**) trials

To unpack the significant interaction between target and bag exposure in Practice, another linear model was run. This time, the target exposure binomial z-score was entered as a categorical variable to allow for an estimate of a slope coefficient for bag exposure at each level of target exposure for hit trials (Table 5) and for correct rejection trials (Table 6). For this model, participants with the two lowest target exposure binomial z-scores (equivalent of participants who never saw a target during the exposure trials or only saw one) were excluded from analysis as it was improbable to see targets this infrequently in Practice, and therefore, there were too few participants in these bins. Headphone exposure was omitted from this model to have sufficient power to describe the interaction between target and bag exposure. Examining the effect of bag exposure across various levels of target exposure shows that the effect of bag is larger at lower levels of exposure to target presence (Fig. 4).

**Table 5:** All factors and interactions and associated statistics in the linear model fitted to hit trials of Practice

Practice: hit trials	Estimate	SE	t-stat	*p*-value
Target (z-score = −0.36)	0.001442	0.003039	0.474493	0.635149
Target (z-score = −0.13)	−0.00442	0.002907	−1.52135	0.128174
Target (z-score = 0.09)	**−0.01144**	**0.002906**	**−3.93629**	**8.28E-05**
Target (z-score = 0.37)	**−0.01721**	**0.003024**	**−5.69058**	**1.27E-08**
Target (z-score = 0.84)	**−0.02729**	**0.003639**	**−7.50002**	**6.39E-14**
Bag	**−0.03895**	**0.008119**	**−4.79753**	**1.61E-06**
Target × Bag (z-score = −0.36)	**0.019507**	**0.008922**	**2.186449**	**0.028783**
Target × Bag (z-score = −0.13)	**0.026395**	**0.008522**	**3.097387**	**0.001952**
Target × Bag (z-score = 0.09)	**0.033857**	**0.008519**	**3.974461**	**7.05E-05**
Target × Bag (z-score = 0.37)	**0.044836**	**0.008871**	**5.053995**	**4.33E-07**
Target × Bag (z-score = 0.84)	**0.043501**	**0.010658**	**4.081512**	**4.48E-05**

**Table 6:** All factors and interactions and associated statistics in the linear model fitted to correct rejection trials of Practice

Practice: correct rejection trials	Estimate	SE	t-stat	*p*-value
Target (z-score = −0.36)	0.01	0.006	1.34	0.18
Target (z-score = −0.13)	**0.02**	**0.005**	**3.90**	**9.59E x 10**^**−5**^
Target (z-score = 0.09)	**0.03**	**0.005**	**5.24**	**1.56 x 10**^**−7**^
Target (z-score = 0.37)	**0.04**	**0.005**	**7.18**	**7.16 x 10**^**−13**^
Target (z-score = 0.84)	**0.05**	**0.01**	**8.50**	**1.85 x 10**^**−17**^
Bag	**−0.03**	**0.02**	**−2.28**	**0.02**
Target × Bag (z-score = −0.36)	−0.01	0.02	−0.48	0.63
Target × Bag (z-score = −0.13)	0.01	0.02	0.65	0.51
Target × Bag (z-score = 0.09)	0.03	0.02	1.89	0.06
Target × Bag (z-score = 0.37)	**0.05**	**0.02**	**3.17**	**1.52 x 10**^**−3**^
Target × Bag (z-score = 0.84)	**0.04**	**0.02**	**2.38**	**0.02**

To provide more insights into this interaction, an exploratory analysis was done whereby the data were broken down by target exposure z-score and a model was fit using bag exposure z-score to predict response times (e.g., using bag exposure z-score to predict response times only for participants who had a target exposure z-score of -.36). The beta weights and corresponding p-values of these models for both hit and correct rejection trials are listed in Table 7. For hit trials, bag exposure was a significant predictor of response times for participants with a target presence exposure z-score of -.36 (*F*(1, 73,321) = 28.23, *p* = 1.08 x 10^**−7**^) and -.14 (*F*(1, 152,470) = 23.65, *p* = 1.15 x 10^**−6**^). For correct rejection trials, bag exposure had a significant negative effect on response times for participants with a target presence exposure z-score of -.36 (*F*(1, 31,417) = 54.57, *p* = 1.53 x 10^**−13**^) and -.14 (*F*(1, 84,042) = 47.36, *p* = 1.53 x 10^**-**12^). However, bag exposure had a significant positive effect on response times for participants with a target presence exposure z-score of.37 (*F*(1, 57,110) = 12.77, *p* = 3.52 x 10^**−4**^).

**Table 7:** Beta weights of Bag Exposure predicting response time for each level of target exposure with associated p-value for hit and correct rejection trials in Practice

Practice trials	Hit trials		Correct rejection trials	
Target exposure z-score	Bag exposure β	*p*-value	Bag exposure β	*p*-value
-.36	**-.02**	**1.08 x 10**^**−7**^	**-.04**	**1.53x 10**^**−13**^
-.14	**-.01**	**1.15x 10**^**−6**^	**−0.02**	**5.94x 10**^**−12**^
.09	-.006	.05	-.005	0.11
.37	.006	.1	**.02**	**0.0003**
.84	.004	.52	.01	0.42

The same categorical model was run on correct rejection trials of Level 1 to unpack the significant interaction found there; however in this model, while the main effects of target exposure (*F*(6, 166775) = 14.90, *p* = 4.07 x 10^**−17**^) and bag exposure (*F*(1, 166780) = 11.30, *p* = 7.76 x 10^**−4**^) were significant, the interaction effects between target and bag exposure were not (*F*(6, 166775) < 1.59, *p* > 0.14).

## Discussion

The human visual system excels at leveraging environmental regularities to enhance performance, as is evident from priming, carryover, and hysteresis effects, as well as the ability to build ensemble representations (e.g., Chetverikov et al., 2017; Hansmann-Roth et al., 2021). These effects are typically explored in terms of task-relevant and -irrelevant information, operationalized by the experimenter. However, given the rich and dynamic nature of visual scenes, it can be challenging to map task relevance to specific features of a scene. Instead, the visual system tracks visual information as it comes in and learns what aspects are correlated with and aid completion of the current task at hand. Understanding this process is a fundamental goal of cognitive science as it lends insights not only into what information the visual system is currently storing but also into how this information flexibly changes through repeated exposure to a task. This also shifts the perspective from studying constructs of an experimental paradigm to understanding how the visual system operates in the real world. The results of the present study demonstrate that, with enough power, it is clear that information across a range of task relevance drives behavior, and moreover, that these different sources of information interact. These findings demonstrate that the human visual system is capable of tracking and optimizing relative to a wealth of complex visual statistics to drive adaptive behavior.

To characterize how repeated task exposure influences performance, previous work used a massive dataset generated from gameplay of the *Airport Scanner* mobile game (Kramer et al., 2022) to examine target exposure, headphone exposure (a salient distractor), and bag type exposure (task-irrelevant, context information), and found that each had an effect on performance (Kramer et al., 2022). The current work built off this finding by combining all three factors into a single omnibus model. The purpose of this was to better understand whether these factors accounted for a unique portion of performance variability, how those effects compared to one another, and whether there existed any interactions between factors.

As with the previous work, target exposure was found to have an effect on response times in both target-present and target-absent trials in the omnibus model. In both Level 1 and Practice data, the effect was response contingent. The more exposure participants had to target-present trials, the faster they were to find a target in target-present trials and the slower they were to correctly reject target-absent trials (~65-ms decrease in response time between the lowest level of target exposure and the highest in hit trials and a ~145-ms increase in correct rejection trials). The robustness of this effect, even when target exposure is entered into the model with other factors, is in line with the bidirectional relationship between selective attention and visual statistical learning (Theeuwes et al., 2022; Turk-Browne et al., 2005), though does not itself speak to causality.

In the omnibus model, increased exposure to a salient distractor (i.e., headphones) decreased response times in target-present trials when that salient distractor was present. One potential explanation for this effect could be found in the learned distractor suppression literature, where a salient distractor, presented over repeated trials, is suppressed and no longer produces an attentional capture effect (Gaspelin & Luck, 2018; Gaspar & McDonald, 2014). Therefore, the speed-up in response times to targets seen when participants have increased exposure to the headphones may be due to a suppression template being implemented, thereby limiting the interference created by the salient distractor. Alternatively, these results also support previous findings that show more efficient search with increased familiarity with distractors (Mcurzek & Sheinlberg, 2005; Richards & Reicher, 1978). However, it is less straightforward how suppression of a salient distractor would affect rejection time in target-absent trials. The current results did not find a significant effect of headphone exposure on response time in target-absent trials in the Level 1 data but did in the practice data. Suppression of a salient distractor has been found even in the absence of a target (Chang & Egeth, 2021) and potentially a speed-up in response times in target-absent trials would be predicted as well. However, it has been shown that the presence of a salient distractor in target-absent trials can induce early quitting and therefore decrease response times (Moher, 2020). More explicitly, this pattern of results could be explained as increased exposure to the salient distractor making them less salient, and therefore less likely to induce early quitting in the Level 1 trials. As such, the lack of a consistent headphones exposure effect seen in target-absent trials does not necessarily go against the salient distractor suppression theory but rather suggests that this suppression does not necessarily have the same benefits on overall performance when the decision is to determine a target is not present and terminate a search since the potential suppression is likely occurring in concert with other factors.

Finally, for the main effects of the putatively least task-relevant factor, increased exposure to the particular bag type present in the performance trial led to decreased response times. This effect was found in both target-present and target-absent trials of Practice data but only in target-absent trials of Level 1. This discrepancy in results could also be because the targets are found too quickly for the bag to have an effect in the later Level 1 data. However, it should also be noted that target-present trials have less variability in response times than target-absent trials and, as a result, might require the increased sample size available in Practice data (800,000 participants) compared to Level 1 data (489,000 participants). These results suggest that the visual system may track the recurrence of task-irrelevant features and this exposure accounts for a unique portion of variance even when incorporated with other more task-relevant exposure factors. This effect is small, however, and may require sufficient power and performance variability to be evident.

Interestingly, the current work reveals an interaction between exposure to bag type and exposure to target presence. This interaction shows that repeated task-irrelevant context information has a greater effect on performance when participants have less exposure to target presence. This result is similar to previous findings which show a change in attentional strategy as participants gain experience with target-present trials (Duncan et al., 2024; Jiang & Chun, 2001; Jiang & Leung, 2005). Potentially, exposure to target-present trials fine tunes one’s attentional set to the features and probable locations of the target (e.g., blue and within the bag), discouraging attention from surrounding bag information. By extension, the interaction no longer being significant in Level 1 could be due to the participant’s general increased experience in the game. It should again be noted though that the sample size was considerably smaller in Level 1 than in Practice, and likely contributed to the lack of effect found in the exploratory model, which required more power as it coded target exposure scores as a discrete, categorical predictor. However, there are other confounding factors to consider. For example, response times are slower in target-absent trials. This extra time could allow participants to attend more to contextual information (i.e., the bag type). Likewise, one could argue that participants hit floor or ceiling performance (depending on whether it is a hit or correct rejection trial) at higher levels of target exposure. However, the exploratory analysis showed that in correct rejection trials there was a small reversal of effect rather than just a disappearance. This suggests that potentially an association is built between the bag type and target-absence so that when the bag is presented without a target (as is the case in correct rejection trials) there is a speed-up in response times. Conversely the more exposure an individual has to a bag containing targets, the slower they are to respond than when the bag is presented without a target. As multiple factors may be contributing to this interaction and the interaction was not seen in hit trials of Level 1, further work is required to disentangle the effect.

Together, these results suggest that the visual system tracks a wide variety of sources of information in repeated visual search tasks, which experimenters should be aware of when developing paradigms. The difficulty in quantifying exactly how the visual system tracks information across a spectrum of task-relevance is that it requires an intense amount of data. Depending on the amount of exposure the experimenter wants to give the participant (e.g., the number of trials participants complete before the performance trial), varying even two factors (e.g., how often a participant sees a target and how often they see a salient distractor) can require a huge number of participants to ensure a sufficient sample at each exposure level for each factor. Classic, in-person data collection rarely yields large enough sample sizes to look at the effects that exposure to various task elements can simultaneously have on performance. The consequence of this is that simplified lab studies can only reveal a subset of the effects, namely, larger ones. However human behavior in real-world scenarios is complex, the product of a multitude of large and small effects, the contribution of which must be understood to understand the system as a whole. Big Data allows the characterization of smaller effect sizes and their complicated interactions. However, as with any data collection at such a scale, with this statistical power comes an increase in variability (e.g., participants play when they like, for as long as they like, in the environment of their choosing) and a limitation on the available data streams (mobile or online data collection more generally will be limited in collecting physiological measures). The complementary aspects of large, sparse data sets and controlled, multimodal, in-lab data collections speak to the strength in combining the two approaches for effective advancement of the field. Towards this aim, the current work seeks to demonstrate the unique contribution that massive datasets can provide to the field, not only in providing insight into what information is learned through repeated task exposure but also in motivating more targeted, smaller-scale studies which can further unpack these effects.

## Data Availability

The data used in this study are owned by Kedlin Company. All requests for the data for research purposes must be made to Kedlin Company through the authors.

## Code availability

Upon publication, all analysis code used in this study will be made available on the same Open Science Framework page where the preregistration is currently posted (https://osf.io/3rka8/).
